# Book Review

**Published:** 1947

**Authors:** 


### Reviews of Books

**Massage and Remedial Exercises.** By Noel M. Tidy. Seventh Edition. Pp. viii., 480. Illustrated. Bristol: John Wright & Sons Ltd. 1947. Price 25s.—There is little to be added to our eulogy in 1944 of the sixth edition of Tidy's *Massage and Remedial Exercises.* There are only a few alterations and additions in this seventh edition. Surprisingly, there is no mention of exercise therapy for aural vertigo. Suspension and pulley exercises might, with advantage, be enlarged upon. But perhaps there is already too much packed into the volume.

**Symptoms and Signs in Clinical Medicine.** By E. Noble Chamberlain, M.D. Fourth Edition. Pp. viii., 463. Illustrated. Bristol: John Wright & Sons Ltd. 1947.—It is difficult to exaggerate the merits of this excellent work. The changes since the third edition have still further improved it. The section, for example, on the central nervous system has been brought up to date and revision appears to have been carefully carried out in other sections of the work. It is as beautifully printed as ever, the illustrations being most splendidly reproduced; and the whole work reflects the greatest credit upon the author, his collaborators and the publishers. It will prove a safe guide to the student commencing medicine; and few who are much more senior will fail to derive some benefit from perusal of its pages.

**Diseases of the Heart and Circulation.** By A. A. F. Peel, D.M., F.R.F.P.S. Pp. xxxi., 398. Illustrated. London: Oxford University Press (Geoffrey Cumberlege). 1947. Price 35s.—This book is based on the author's lectures delivered to medical students at the Anderson College of Medicine, Glasgow. As such it is an interesting exposition of orthodox teaching, but can hardly be regarded as a text-book of cardiology, as no attempt is made to cover the whole field. The attention paid to the different facets of cardio-vascular diseases, while no doubt reflecting the author's interests, has not been determined by their relative importance or frequency. Although designed on a physiological basis, no attempt is made to discuss recent views and theories on the mechanism of the different types of circulatory failure, and some of the more recent experimental methods of investigation have been rather neglected. Despite these criticisms this work does form a sound introduction to the study of diseases of the cardio-vascular system. It has been beautifully produced with lavish illustrations, and the whole production is reminiscent of pre-war publications.

**Common Skin Diseases.** By A. C. Roxburgh, M.D., F.R.C.P. Forty-eighth Edition. Pp. xxxii., 498. Illustrated. London: H. K. Lewis & Co. Ltd. 1947. Price 21s.—It is a pleasure to see a further edition of Dr. Roxburgh's excellent text-book, which has always been popular with medical practitioners and students since its first publication in 1932. In spite of war-time difficulties, four new editions and five reprints were issued between September, 1939, and the present time, to meet the demand for this book. There are a number of additions, notably the uses in dermatology of penicillin and D.D.T., and recent experiences with sulphonamides, including their dangers when applied externally. Certain diseases have been added, such as adenoma sebaceum, erysipeloid, meadow dermatitis, ticks, and jelly-fish stings, which the author considers to be " common." The section on lupus vulgaris has been brought up to date by the inclusion of the calciferol treatment, recently introduced in this country by Dowling and Prosser Thomas. The chapter dealing with varicose veins, eczema and ulcer, has been re-written with the help of a surgical colleague, with the object of helping practitioners to decide which of their cases are suitable for operative treatment and which are not, and how to treat each type of case. Eight coloured plates are included. These are improved by viewing through a pale-blue glass as the reproductions appear to lack blue. The " violaceous " colour of lichen planus (plate VII, page 385) is much improved by this method, and it is hoped that it will be possible to incorporate more blue in colour-printing in the future. There are 212 black and white illustrations and these, on the whole, are excellent. Dr. Roxburgh is a keen and very able photographer; by taking his own photographs he can ensure that the particular characteristics of a skin lesion which he wishes to demonstrate are shown. The index of preliminary diagnosis is retained in this edition. This is used by ascertaining the nature of the lesions of which an eruption is composed, then referring to the index one finds a number of diseases (with the page numbers) listed under the particular type of lesion. After reading the descriptions in the text of the most probable of these diseases, a final diagnosis is made. This text-book can be confidently recommended to the general practitioner and student alike.

**Diseases of the Nervous System.** By W. RUSSELL BRAIN, D.M., F.R.C.P. Third Edition. Pp. xx., 987. Illustrated. London: Oxford University Press (Geoffrey Cumberlege). 1947. Price 37s. 6d.—A third edition of this valuable text-book appears after an interval of seven years. Much rearrangement and numerous additions have been found necessary. The rapid advances in neurology conditioned by war problems, and by the advent of chemotherapy and penicillin in particular, are obvious. The whole outlook with regard to prognosis is changed. The war produced abundant clinical material for the study of injuries; but it also brought into prominence a vast field of medical disorders, infective and nutritional. The general arrangement of the book remains unaltered. The early chapters are devoted to anatomy and physiology. Under the latter heading it is to be regretted that electroencephalography is not allotted more space and discussion. The average reader looks for a discussion of physiological principles and is interested in an appraisement of the clinical value of the method. He has little time or inclination to delve into original monographs of complicated technology. The brief references, the illustrations of tracings and descriptions from an abstract by Grey Walker and Dovey are incomprehensible as they stand. The chapter on demyelinating diseases of the nervous system is of particular value, coming as it does with the great authority of the author. The subject has a particular interest at the present time in view of recent research on sway back in lambs. The sciaticas and brachial neuralgias come in for review in the light of their surgical associations. Ancillary methods of diagnosis, elaborated on the basis of modern radiological technique, have necessitated much rewriting. Angiography seems likely to be more widely used since opaque iodine compounds have replaced thorotrast. Much more space has been devoted in the new edition to the psychological aspects of neurology. As a general text-book of neurology this volume meets the requirements of the general physician and neurologist. It is a book to possess.

**Clinical Examination of the Nervous System.** By G. H. Monrad-Krohn, M.D., F.R.C.P. Eighth Edition. Pp. xx., 380. Illustrated. London: H. K. Lewis & Co. Ltd. 1947. Price 16s.—This book, first published in 1921, has now reached an eighth edition which speaks for itself. Throughout a period of twenty-five years it has met with the approval of neurological workers in England, Norway and other countries. It is not intended to be a text-book of neurology; but it occupies a position comparable, perhaps, to Hutchison's *Clinical Methods*. In its own sphere it contains much detailed information, based on applied physiology. The discussion of clinical syndromes is essentially anatomical and physiological. Aetiology is not directly considered. The value of the book is to be found in its clear description and assessment of physical signs, together with an exposition of the physiological principles on which they rest. The writer stresses the importance of psychosomatic disorders. There is an illuminating chapter on sense perception, under the heading "psychosomatic examination." Towards the end of the book much space is devoted to modern ancillary methods of investigation, such as air-studies and angiography, the whole generously illustrated. It is disappointing to find only a passing reference to electroencephalography, which is dismissed with the advice that it had better be left to specially trained neuro-physiologists. While the author has obviously avoided the subject on purpose, the clinician needs readily available information on the general principles of a method, at present little understood, and as to the useful scope of which there is much misconception.

**Pye's Surgical Handicraft.** Edited by Hamilton Bailey, F.R.C.S. Fifteenth Edition. Pp. xii., 668. Illustrated. Bristol: John Wright & Sons Ltd. 1947. Price 25s.—This valuable and well-established work has been further enlarged by the inclusion of yet more diagrams and illustrations and two new chapters. One of these is concerned with penicillin and the other with the treatment of wounds and compound fractures. Only minor points of criticism can be made in this otherwise most comprehensive and compact work. In the section on burns the use of the radiant heat cradle is referred to for shock. It is open to question as to whether it is desirable to bring about vaso-dilatation of surface vessels, when there is some evidence to suggest that the vaso-constriction is a protective compensatory mechanism to combat a low pressure and diminished blood volume. Plasma transfusion is included and rather lost in a section entitled "Fluids." The early and rapid adjustment of the blood chemistry in burns is more important than giving drinks to a patient who is often actually vomiting. No mention is made of jaundice following upon the use of pooled plasma, and it is doubtful whether it is even worth while mentioning any coagulent methods of treating burns. On page 366 the incisions illustrated for draining tendon sheaths are not those recommended by everyone these days. Nevertheless, this is a most useful book and fills a large gap in routine teaching, and should be most valuable to all housemen. It is eminently practical, well set out and probably is among the best illustrated surgical text-books of the English-speaking world.

**Health Services in England.** By R. C. WOFINDEN, M.D. Pp. 191. Bristol: John Wright & Sons Ltd. 1947.—Dr. Wofinden has produced a well-written and stimulating account of a subject which can make dull reading unless it is presented in an interesting fashion. This interest has been maintained throughout partly by the historical approach and also by the obvious sincerity and enthusiasm of the writer. The subject-matter is up to date and covers a wide range of subjects; although there is no systematic list of references, there are frequent references in the text to important books and articles. These are annotated at the foot of the page and there is an adequate index. The standard of printing and general layout of the book are consistently good, and the absence of any illustrations has enabled the book to be produced in a compact and readable form.

